# Computed Tomography Lung Density Analysis: An Imaging Biomarker Predicting Physical Inactivity in Chronic Obstructive Pulmonary Disease: A Pilot Study

**DOI:** 10.3390/jcm12082959

**Published:** 2023-04-19

**Authors:** Yoriyuki Murata, Tsunahiko Hirano, Keiko Doi, Ayumi Fukatsu-Chikumoto, Kazuki Hamada, Keiji Oishi, Tomoyuki Kakugawa, Masafumi Yano, Kazuto Matsunaga

**Affiliations:** 1 Department of Respiratory Medicine and Infectious Disease, Graduate School of Medicine, Yamaguchi University, Ube 755-8505, Japan; yomurata@yamaguchi-u.ac.jp (Y.M.); decem119@yamaguchi-u.ac.jp (K.D.); chiku05@yamaguchi-u.ac.jp (A.F.-C.); khamada@yamaguchi-u.ac.jp (K.H.); ohishk@yamaguchi-u.ac.jp (K.O.); kazmatsu@yamaguchi-u.ac.jp (K.M.); 2 Department of Pulmonology and Gerontology, Graduate School of Medicine, Yamaguchi University, Ube 755-8505, Japan; kakugawa@yamaguchi-u.ac.jp; 3 Department of Medicine and Clinical Science, Graduate School of Medicine, Yamaguchi University, Ube 755-8505, Japan; yanoma@yamaguchi-u.ac.jp

**Keywords:** physical activity, sedentary behavior, density analysis

## Abstract

Physical inactivity correlates with poor prognosis in chronic obstructive pulmonary disease (COPD) and is suggested to be related to lung hyperinflation. We examined the association between physical activity and the expiratory to inspiratory (E/I) ratio of mean lung density (MLD), the imaging biomarker of resting lung hyperinflation. COPD patients (n = 41) and healthy controls (n = 12) underwent assessment of pulmonary function and physical activity with an accelerometer, as well as computed tomography at full inspiration and expiration. E/I_MLD_ was calculated by measuring inspiratory and expiratory MLD. Exercise (EX) was defined as metabolic equivalents × duration (hours). COPD patients had higher E/I_MLD_ (0.975 vs. 0.964) than healthy subjects. When dividing COPD patients into sedentary (EX < 1.5) and non-sedentary (EX ≥ 1.5) groups, E/I_MLD_ in the sedentary group was statistically higher than that in the non-sedentary group (0.983 vs. 0.972). E/I_MLD_ > 0.980 was a good predictor of sedentary behavior in COPD (sensitivity, 0.815; specificity, 0.714). Multivariate analysis showed that E/I_MLD_ was associated with sedentary behavior (odds ratio, 0.39; *p* = 0.04), independent of age, symptomology, airflow obstruction, and pulmonary diffusion. In conclusion, higher E/I_MLD_ scores are associated with sedentary behavior and can be a useful imaging biomarker for the early detection of physical inactivity in COPD.

## 1. Introduction

Physical activity (PA) in patients with chronic obstructive pulmonary disease (COPD) is lower than that in healthy subjects [1], and this decline is associated with a greater risk of mortality and hospitalization [2,3,4]. PA in patients with COPD is related to many factors, including age, sex, and the BODE index, which synthesizes information on body mass index, degree of airflow obstruction, dyspnea, and exercise (EX) capacity [5].

PA and EX tolerance in patients with COPD have previously been evaluated using self-administered questionnaires, step counts per day, and evaluations of the 6 min walking distance (6MWD). Of late, triaxial accelerometers have been more sensitive predictors of mortality and hospitalization [3,4]; however, PA evaluation with this method necessitates a dedicated measuring device and reliable measurement data recorded on at least three dry (no rain) weekdays [6]. Therefore, this evaluation method is not practical for all patients with COPD.

Physical inactivity has been observed even in the early stages of COPD, before the onset of breathlessness [7]. Recently, the usefulness of inspiratory–expiratory computed tomography (CT) has been reported in patients with COPD [8]. Specifically, the expiratory to inspiratory (E/I) ratio of the mean lung density (MLD; E/I_MLD_), an imaging biomarker, has been reportedly used for the evaluation of small airway disease [9,10,11]. E/I_MLD_ has been associated with pulmonary function [10,11,12] and EX tolerance [13]; moreover, it can detect lung hyperinflation, measured as residual volume (RV)/total lung capacity (TLC), more accurately than can the change in relative lung volume, with attenuation values between −860 and −950 Hounsfield units (HU) [14].

In COPD patients, airflow obstruction occurs due to airway wall inflammation and thickening due to inflammatory cell infiltration [15,16]. The resulting air trapping and lung hyperinflation, which are characteristic features of COPD, are more strongly associated with decreased PA than is airflow obstruction [17,18,19], and reports suggest that these two factors contribute to the vicious cycles of COPD aggravation [20]. If lung hyperinflation is closely associated with the mechanisms of physical inactivity, then E/I_MLD_ may be useful for detecting physical inactivity and predicting poor prognoses in COPD, given that CT is commonly performed for the assessment of patients with COPD.

Therefore, the aim of the present study was to evaluate the association between PA and E/I_MLD_ and to examine the ability of imaging biomarkers to detect physical inactivity in patients with COPD. The hypothesis was that E/I_MLD_ is associated with physical inactivity in COPD. To the best of our knowledge, no prior studies have evaluated this association.

## 2. Materials and Methods

### 2.1. Study Design and Subjects

Between 2016 and 2020, ambulatory patients with COPD aged > 40 years at the Yamaguchi University Hospital were recruited for this cross-sectional prospective study. We did not perform power calculations to determine the optimal sample size for statistical significance because this was an exploratory study investigating the association between lung density analysis and PA in a small enrolled sample. We also recruited 12 healthy controls, aged > 40 years, who underwent CT for further evaluation of abnormal findings detected during medical examinations and did not demonstrate any abnormalities on the CT images. All participants underwent pulmonary function tests. COPD was diagnosed according to a post bronchodilator forced expiratory volume in a 1 s/forced vital capacity (FEV_1_/FVC) ratio of < 70%. The exclusion criteria were as follows: poor disease control; presence of other diseases that could affect walking, such as lower limb paralysis; requirement of long-term oxygen therapy; and presence of malignant tumors, which can restrict PA. All patients were stable and had not experienced exacerbation for at least 4 weeks. The symptomology was evaluated using the COPD Assessment Test (CAT) and the Modified Medical Research Council (mMRC) Dyspnea Scale. All participants received an explanation of the study and provided written informed consent prior to participation. The study was approved by the institutional review board (No. H27-204-3) of Yamaguchi University Hospital and has been registered in the UMIN Clinical Trials Registry (UMIN 000024749). The protocol was in accordance with the principles of the Declaration of Helsinki and its later amendments.

### 2.2. PA Evaluations

PA was measured using an accelerometer (Active Style Pro HJA-750C; OMRON HEALTHCARE Co., Ltd., Kyoto, Japan). Although this device is small (30 × 52 × 12 mm) and lightweight (approximately 23 g), it can effectively estimate metabolic equivalents (METs) every 10 s using an internal triaxial accelerometer. All participants wore this device during all waking hours for 2 consecutive weeks. Their PA levels, denoted as EX values, were defined as METs multiplied by their durations (i.e., METs × hour/day) during the last 3 days of monitoring (excluding rainy days and holidays), according to the methodology described in previous studies [6,21]. The duration of each PA with a value of > 1–4 METs was measured in minutes. Patients with COPD showing EX values of < 1.5 (equivalent to < 30 min of walking time per day) and those showing EX values of ≥ 1.5 were divided into the sedentary and non-sedentary groups, respectively [22,23].

### 2.3. CT Scanning and Lung Density Analysis

All participants underwent volumetric chest CT scans at full inspiration, and expiration was measured using a scanner (Aquilion 64, Toshiba Medical Systems, Otawara, Japan) with the following parameters: 120 kVp; thickness, 1 mm; and rotation time, 0.28–0.5 s. Density analyses were performed using a commercial workstation (Virtual Place; AZE Inc., Tokyo, Japan), as described below. The average HU value for the total lung density area (−1000 to −300 HU) on CT conducted at inspiration was calculated as the inspiratory MLD (I). Expiratory MLD (E) was determined at expiration. E/I_MLD_ was denoted as the E/I ratio [24]. An increase in the E/I ratio, which occurs when there is little difference in HU between inspiration and expiration, indicates the presence of more severe lung hyperinflation and air trapping [9,12,14]. A low attenuation area (LAA) was defined as an area with an attenuation value of < –950 HU in the lung parenchyma.

### 2.4. Statistical Analyses

All statistical analyses were performed using EZR statistical software (version 1.40; Saitama Medical Center, Jichi Medical University, Shimotsuke, Japan); a modified version of the R commander that was designed to add statistical functions (The R Project for Statistical Computing, Vienna, Austria) [25]. Continuous variables are presented as medians ± interquartile ranges, and categorical variables are presented as numbers and percentages, as appropriate. Comparisons between two continuous variables were performed using the Mann–Whitney U test, whereas those between two categorical variables were performed using Fisher’s exact test. Spearman’s rank correlation analysis was performed to detect the correlations between E/I_MLD_ and various EX parameters. To identify the predictive factors for sedentary behavior in COPD, we used multivariate logistic regression analysis to calculate adjusted odds ratios (ORs) and the associated 95% confidence intervals (CIs). Variables that reportedly affect PA, including age, CAT score, percent predicted FEV_1_ (%FEV_1_), and percent predicted carbon monoxide diffusing capacity (%DLCO), were included in the multivariate model [5]. A two-side *p*-value of < 0.05 was considered statistically significant.

## 3. Results

### 3.1. Healthy Subjects vs. Patients with COPD

**Figure 1:**
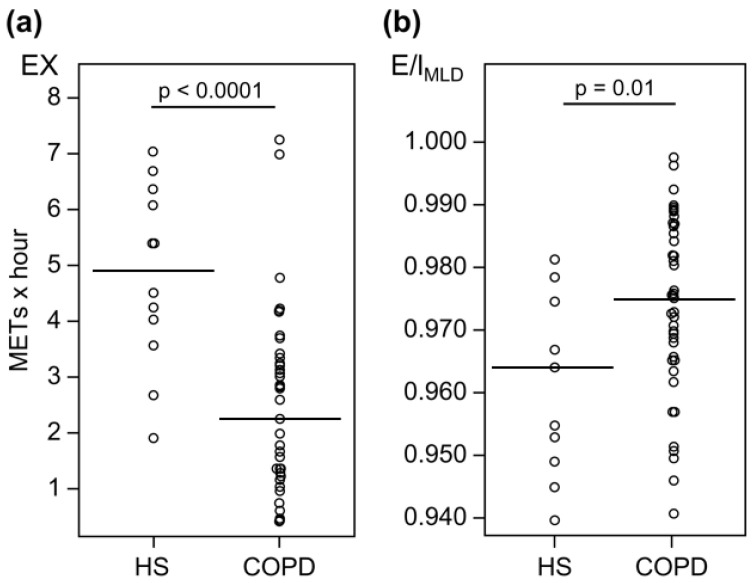
Comparison of EX (**a**) and E/I_MLD_ (**b**) values between healthy subjects and patients with COPD. EX values are lower (median, 2.29 vs. 4.97 METs × hour; *p* < 0.0001) (**a**); while E/I_MLD_ values are higher (median, 0.975 vs. 0.964; *p* = 0.01) for patients with COPD than for healthy subjects (**b**). Definition of abbreviations: HS, healthy subjects; COPD, chronic obstructive pulmonary disease; METs, metabolic equivalents; EX, exercise (METs × hour); E/I_MLD_, expiratory to inspiratory ratio of the mean lung density. The horizontal bars indicate median values.

**Figure 2:**
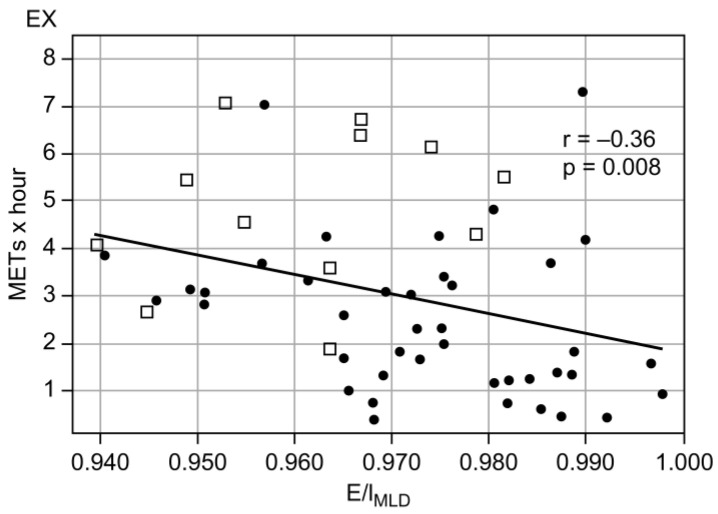
Correlations between EX and E/I_MLD_ values for healthy subjects and patients with COPD. □, healthy subjects; ●, patients with COPD. Definition of abbreviations: COPD, chronic obstructive pulmonary disease; METs, metabolic equivalents; EX, exercise (METs × hour); E/I_MLD_, expiratory to inspiratory ratio of the mean lung density.

The baseline characteristics of the participants are shown in Table 1. We enrolled 12 healthy controls and 41 patients with stable COPD. Compared with healthy subjects, patients with COPD were older and had higher CAT scores. Values of pulmonary function parameters such as FEV_1_ and FVC were also significantly lower in patients with COPD than in healthy subjects. Among the patients with COPD, 19, 20, and 2 showed stages 1, 2, and 3 COPD, respectively, according to the Global Initiative for Chronic Obstructive Lung Disease (GOLD) criteria. Thus, almost all the enrolled patients presented with mild-to-moderate COPD. COPD patients with mMRC ≥ 2 exhibited worsening airflow obstruction, hyperinflation, and pulmonary diffusion compared with healthy subjects (Table S1). The EX values were significantly lower (median, 2.29 vs. 4.97 METs × hour; *p* < 0.0001), while E/I_MLD_ values were significantly higher (0.975 vs. 0.964, *p* = 0.01) for patients with COPD than for healthy subjects (Figure 1). E/I_MLD_ was significantly correlated with EX for all participants (patients with COPD and healthy subjects: Figure 2, r = –0.36, *p* = 0.008) and for patients with COPD (Table 2, r = –0.32, *p* = 0.04), but not for healthy subjects (Table 2, r = –0.32, *p* = 0.32). Moreover, E/I_MLD_ showed a significant negative correlation with the duration of higher intensity EX (e.g., > 3 METs; Table 2).

### 3.2. Predictive Factors for Detecting Sedentary Behavior in COPD

**Figure 3:**
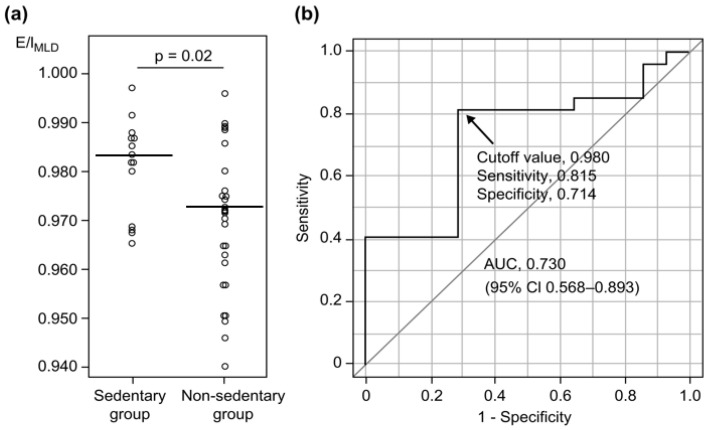
Comparison of E/I_MLD_ for physical activity (**a**) and ROC curve evaluation of the utility of E/I_MLD_ for detecting sedentary behavior in patients with COPD (**b**). A comparison of E/I_MLD_ values between the sedentary (patients with EX values of < 1.5, n = 14) and nonsedentary (patients with EX values of ≥1.5, n = 27) groups demonstrates a statistically significant between-group difference (median, 0.983 vs. 0.972; *p* = 0.02). The ROC evaluation of the utility of E/I_MLD_ for detecting sedentary behavior in patients with COPD is shown here. If the cutoff value of E/I_MLD_ is 0.980 (arrow), the sensitivity and specificity are 0.815 and 0.714, respectively. The AUC is 0.730 (95% confidence interval, 0.568–0.893). Definition of abbreviations: E/I_MLD_, expiratory to inspiratory ratio of the mean lung density; ROC, receiver operating characteristic; COPD, chronic obstructive pulmonary disease; EX, exercise; AUC, area under the receiver operating characteristic curve. The horizontal bars indicate median values.

Table 3 shows the results of comparisons between the sedentary and nonsedentary groups of COPD patients. E/I_MLD_ was significantly higher in the sedentary group than in the nonsedentary group (0.983 vs. 0.972, *p* = 0.02; Table 3, Figure 3a). However, CAT scores and pulmonary function parameters, including FEV_1_, FVC, DLCO, and LAA%, were not significantly different between the groups, even after adjustments for age, smoking index, and the mMRC Dyspnea Scale score (Table 3). Moreover, an E/I_MLD_ of > 0.980 exhibited a sensitivity of 0.815 and a specificity of 0.714 (area under the receiver operating characteristic [ROC] curve [AUC] = 0.730) (Figure 3b) for the identification of sedentary patients with COPD, whereas an age of > 71 years yielded a sensitivity of 0.704 and a specificity of 0.786 (AUC = 0.745; 95% CI, 0.568–0.922).

The results of multivariate analysis indicated that E/I_MLD_ was significantly associated with sedentary behavior (EX < 1.5) in patients with COPD, with an adjusted OR of 0.39 (95% CI, 0.16–0.95; *p* = 0.04) when accounting for age, CAT score, predicted %FEV_1_, and %DLCO (Table 4).

## 4. Discussion

The present study demonstrated that higher E/I_MLD_ values were associated with physical inactivity in stable COPD patients. To the best of our knowledge, this is the first study to demonstrate an association between E/I_MLD_ values and PA in patients with COPD. We also found that a higher E/I_MLD_ value (>0.980) was a predictive factor for physical inactivity in patients with COPD, irrespective of age, symptomology, airflow obstruction, and pulmonary diffusion. These results confirm that higher E/I_MLD_ values, which are associated with air trapping and lung hyperinflation, can be useful for detecting sedentary behavior in patients with COPD. This suggests that E/I_MLD_ can be a new tool for evaluating physical inactivity in patients with COPD (as an alternative to triaxial accelerometers). We showed that E/I_MLD_ evaluated using inspiratory and expiratory CT readings showed a significant negative correlation with PA (Figure 2, Table 2). We also detected sedentary behavior in patients with COPD (Figure 3). In a previous study, E/I_MLD_ was elevated in smokers with predicted FEV_1_ values of < 80% and/or DLCO values of < 80% [10]. Another study evaluating patients with emphysema showed that E/I_MLD_ was negatively correlated with the predicted %FEV_1_ in patients with COPD (in contrast to %LAA) [11]. Studies have also shown that E/I_MLD_ values are more strongly associated with air trapping derived from the RV to TLC ratio (RV/TLC) [9,12] and positively correlated with small airway obstruction evaluated by a single-breath nitrogen test [26]. These findings indicate that E/I_MLD_ may be an effective imaging biomarker for detecting air trapping, airflow obstruction, and small airway obstruction in smokers and patients with COPD. This may explain the association between E/I_MLD_ and EX tolerance observed in the previous study [13], as well as the association between E/I_MLD_ and physical inactivity in the present study.

Interestingly, the present study demonstrated that sedentary patients with COPD tended to exhibit mild dyspnea (predominantly mMRC grade 0–1, 71.4%; Table 3). Physical inactivity is highly prevalent, even in patients with COPD who are not aware of severe dyspnea (mMRC grade 0, 45.8%; grade 1, 47.2%) [27], and symptom questionnaires may therefore underestimate physical inactivity in patients with COPD [28]. Similarly, the present study showed that the evaluation of symptoms on the basis of mMRC and CAT scores is not necessarily useful for the earlier detection of physical inactivity in patients with COPD. In addition to the significant negative correlation between E/I_MLD_ and PA in patients with COPD (Figure 2, Table 2), a strong correlation between higher EX intensity and E/I_MLD_ was observed in the present study (r = –0.15 for > 2 METs, –0.31 for > 3 METs, –0.36 for > 4 METs) (Table 2). Minakata et al. reported that PA of higher intensity was reduced in patients with COPD; compared with healthy subjects, patients with COPD showed reductions of 23.1% for ≥ 2.0 METs and 66.9% for ≥ 3.5 METs [29]. This report helps explain why E/I_MLD_ preferentially identifies a decrease in PA of significantly higher intensity.

The PA levels of patients with COPD enrolled in the present study were significantly lower than those of healthy subjects, although the majority of enrolled subjects had mild-to-moderate COPD, and the median predicted FEV_1_ in patients with COPD was 77.0% (Table 1). Moreover, half the sedentary patients with COPD had GOLD stage 1 COPD (14/27, 52%; Table 3). Previous studies have shown that FEV_1_, inspiratory capacity (IC), RV, and DLCO values are associated with PA in patients with COPD [17,18,19]. However, our data showed that the values of various pulmonary function parameters, such as airway obstruction (FEV_1_), static hyperinflation (IC/TLC), and diffusing capacity (DLCO), were not significantly different between sedentary and nonsedentary patients with COPD (Table 3). These differences may be attributed to the varying backgrounds of the participants. For example, in a previous study that included patients evenly categorized according to GOLD stages 1–4, the mean predicted %FEV_1_ was 40–60%, which was significantly lower than that in the present study [17,18,19]. In other words, prior studies have shown that airway obstruction is associated with physical inactivity in patients with more severe COPD, whereas the present study indicated that E/I_MLD_ is more useful than is spirometry for detecting physical inactivity in patients with less advanced COPD. Further prospective studies are required for a more comprehensive evaluation of the association between PA and lung density.

Of late, CT evaluations have shown that extrapulmonary factors such as skeletal muscles are associated with PA and air trapping [30]. Because E/I_MLD_ is the difference between inspiration and expiration on CT, it may be affected by the strength of the lungs and respiratory muscles. Additional studies should evaluate the association between respiratory muscle volume and density in sedentary patients with COPD in more detail. In the present study, ROC curve analyses indicated that an E/I_MLD_ value of > 0.980 showed good sensitivity with a high AUC value for the detection of sedentary behavior in patients with COPD (Figure 3b). Of course, age was correlated with both physical activity and E/I_MLD_ because age was thought to effect physical activity and progressive hyperinflation in COPD patients. Nevertheless, multivariate analyses showed that the association between E/I_MLD_ and sedentary behavior in patients with COPD was independent of age, CAT score, % predicted FEV_1_, and %DLCO (Table 4). Our findings indicate that E/I_MLD_ is useful to screen for physical inactivity prior to symptom progression and onset of lung dysfunction in COPD. Aging was a predictive factor for physical inactivity, but sensitivity was better for E/I_MLD_ than for age. Moreover, the usefulness of bronchodilators for improving PA when evaluating improvements in airway obstruction with COPD has been controversial, as mentioned in a few previous reports [31,32,33,34,35,36,37,38]. In contrast, Takahashi et al. recently reported that tiotropium/olodaterol reduced the duration of PA with 1.0–1.5 METs, thus improving PA [39]. Furthermore, according to the American Thoracic Society guidelines, patients with COPD showing EX intolerance must be treated with long-acting β2-agonist (LABA)/long-acting muscarinic antagonist (LAMA) combination therapy instead of LABA or LAMA monotherapy [40]. This suggests that early detection of physical inactivity may facilitate initial dual bronchodilator treatments to improve PA in inhaler-naive patients with COPD. Other therapeutic interventions, such as long-lasting rehabilitation programs and educational initiatives aimed at increasing PA and thereby optimizing health, also exist [31,41,42]. Moreover, early detection of physical inactivity by the evaluation of E/I_MLD_ may decrease the risk of exacerbation and improve the prognosis of patients with COPD. Although CT increases medical radiation exposure, evaluation of E/I_MLD_ should be considered for patients with COPD who exhibit few symptoms and preserved pulmonary function.

This study has some limitations. First, this was a cross-sectional study conducted at a single institution, and the number of enrolled patients was small. This may have resulted in uncontrolled bias inherent to the study design. Second, most of the targeted patients presented with mild-to-moderate disease, which limits the generalizability of the study findings. Notably, however, the rate of coexistence of severe COPD with heart disease or neuromuscular disorders causing activity restriction is high. Third, certain cardiorespiratory variables that may affect sedentary behavior, such as respiratory muscle force, lung elasticity, and various physiological responses (heart rate, maximal oxygen assumption, and ventilation at maximal EX), could not be accounted for in the present study. Four, all COPD patients were male compared to the female patients in the healthy subjects group (Table 1); therefore, the comparison by sex is not sufficient in this study. The sex differences in COPD patients have been reported to affect dyspnea [43], the severity of CT emphysema [44], and physical inactivity [45]. Future studies should evaluate these topics in a more comprehensive fashion.

## 5. Conclusions

We demonstrated that higher E/I_MLD_ values were associated with sedentary behavior in patients with mild-to-moderate COPD. The results suggest that this imaging biomarker is a useful tool for the early detection of physical inactivity in patients with COPD, which can improve the quality of life and health trajectories in this patient population. Our findings need to be verified in future highly powered investigations. However, they are expected to guide future study directions and, if confirmed, will ultimately facilitate the development of medical guidelines and interventions. 

## Figures and Tables

**Table 1:** Clinical characteristics of healthy subjects and patients with COPD.

	HS (n = 12)	COPD (n = 41)	*p*-Value
Sex (M/F)	6/6	41/0	<0.0001
Age (year)	62 (56–70)	71 (67–74)	0.02
BMI (kg/m^2^)	21.4 (19.8–23.8)	22.8 (20.4–24.4)	0.33
Smoking index (pack-year)	10 (0.0–31)	45 (29–103)	0.0004
CAT	4 (3.5–6.3)	11 (7.8–14.5)	0.001
mMRC Dyspnea Scale (0/1/2/3/4)	6/6/0/0/0	17/15/5/3/0	0.62
FEV_1_ (L)	2.65 (2.39–3.16)	2.21 (1.90–2.46)	0.002
FEV_1_/FVC (%)	78.2 (75.8–86.9)	63.6 (58.0–66.4)	<0.0001
FEV_1_ % pred (%)	110 (103–115)	77.0 (65.9–85.6)	<0.0001
GOLD stage (1/2/3/4)	-	19/20/2/0	-
FVC % pred (%)	110 (105–120)	103 (88.6–118)	0.08
RV % pred (%)	114 (103–117)	105 (98.7–128)	0.84
RV/TLC % pred (%)	100 (90.7–113)	90.7 (80.7–102)	0.46
IC/TLC (%)	44.4 (42.5–49.6)	41.4 (34.1–45.8)	0.18
%DLCO (%)	105 (96.8–125)	90.1 (75.8–109)	0.09
%DLCO/VA (%)	94.6 (89.9–105)	75.0 (65.1–102)	0.18

Data are presented as medians (interquartile ranges). Definition of abbreviations: HS, healthy subjects; COPD, chronic obstructive pulmonary disease; BMI, body mass index; CAT, COPD Assessment Test; mMRC, Modified Medical Research Council; GOLD, Global Initiative for Chronic Obstructive Lung Disease; FEV_1_, forced expiratory volume in 1 s; FVC, forced vital capacity; RV, residual volume; TLC, total lung capacity; IC, inspiratory capacity; DLCO, carbon monoxide diffusing capacity; VA, alveolar volume.

**Table 2:** Coefficients of correlation between exercise intensity and E/I_MLD_ for healthy subjects and patients with COPD.

	E/I_MLD_		
	All Subjects (n = 53)	Healthy (n = 12)	COPD (n = 41)
>1 MET duration (min)	−0.18	−0.02	−0.03
>2 MET duration (min)	−0.21	−0.31	−0.15
>3 MET duration (min)	−0.37 ^†^	−0.31	−0.31 *
>4 MET duration (min)	−0.37 ^†^	−0.46	−0.36 *
Exercise (METs × hour)	−0.36 ^†^	−0.32	−0.32 *

* *p* < 0.05, ^†^ *p* < 0.01. Data are presented as correlation coefficients. Definition of abbreviations: HS, healthy subjects; COPD, chronic obstructive pulmonary disease; METs, metabolic equivalents; E/I_MLD_, expiratory to inspiratory ratio of the mean lung density.

**Table 3:** Comparison between sedentary and nonsedentary groups of patients with COPD.

	Sedentary Group (n = 14)	Nonsedentary Group (n = 27)	*p*-Value
Sex (M/F)	14/0	27/0	-
Age (years)	75.5 (72.0–80.8)	68.0 (66.0–72.o)	0.01
BMI (kg/m^2^)	24.1 (22.0–24.7)	22.7 (20.4–23.9)	0.42
Smoking index (pack-year)	52.9 (41.3–61.5)	38.0 (22.8–51.5)	0.04
CAT	9.5 (7.0–11.8)	12.0 (9.3–16.0)	0.16
mMRC Dyspnea Scale (0/1/2/3/4)	3/7/4/0/0	14/8/1/3/0	0.02
FEV_1_/FVC (%)	61.6 (58.1–66.2)	63.7 (58.5–66.8)	0.69
FEV_1_ % pred (%)	74.4 (58.6–83.8)	80.9 (69.8–90.2)	0.26
GOLD stage (1/2/3/4)	5/9/0/0	14/11/2/0	0.33
FVC % pred (%)	98.0 (80.6–121)	104 (93.3–113)	0.67
RV % pred (%)	115 (103–132)	104 (96.1–121)	0.34
RV/TLC % pred (%)	94.3 (81.2–107)	88.8 (80.7–102)	0.87
IC/TLC (%)	39.0 (33.5–41.8)	44.3 (35.8–47.4)	0.10
%DLCO (%)	92.2 (76.3–109)	89.5 (76.2–109)	0.90
%DLCO/VA (%)	68.0 (60.6–85.8)	85.1 (70.1–107)	0.11
Step per hour	59.1 (49.0–61.5)	64.4 (52.9–73.3)	0.17
6MWD (m)	386 (375–417)	412 (372–459)	0.21
LAA (%)	22.2 (20.9–22.8)	22.5 (20.9–25.6)	0.75
E/I_MLD_	0.983 (0.972–0.987)	0.972 (0.959–0.976)	0.02

Data are presented as medians (IQR, interquartile ranges). Definition of abbreviations: COPD, chronic obstructive pulmonary disease; BMI, body mass index; CAT, COPD Assessment Test; mMRC, Modified Medical Research Council; FEV_1_, forced expiratory volume in 1 s; FVC, forced vital capacity; RV, residual volume; TLC, total lung capacity; IC, inspiratory capacity; DLCO, carbon monoxide diffusing capacity; VA, alveolar volume; 6MWD, 6-min walking distance; SpO_2_, peripheral oxygen saturation; MWT, 6-min walking test; LAA, low attenuation area; E/I_MLD_, expiratory to inspiratory ratio of the mean lung density.

**Table 4:** Univariate and multivariate analyses of factors predicting sedentary behavior in patients with COPD.

Variable	Univariate Analysis		Multivariate Analysis	
	OR (95% CI)	*p*-Value	OR (95% CI)	*p*-Value
Age (years)	0.89 (0.80–0.99)	0.03	0.87 (0.75–1.00)	0.05
CAT	1.05 (0.95–1.15)	0.34	1.15 (0.99–1.33)	0.07
FEV_1_ % pred (%)	1.02 (0.98–1.05)	0.38	1.04 (0.92–1.10)	0.21
%DLCO (%)	1.00 (0.98–1.03)	0.78	0.96 (0.92–1.01)	0.11
E/I_MLD_ (%)	0.47 (0.25–0.87)	0.02	0.39 (0.16–0.95)	0.04

Definition of abbreviations: COPD, chronic obstructive pulmonary disease; OR, odds ratio; CI, confidence interval; CAT, COPD Assessment Test; FEV_1_, forced expiratory volume in 1 s; DLCO, carbon monoxide diffusing capacity; E/I_MLD_, expiratory to inspiratory ratio of the mean lung density.

## Data Availability

The data analyzed during the current study are included in this article. Additional data are available by request to the corresponding author.

## Supplementary Materials

The following supporting information can be downloaded at: https://www.mdpi.com/article/10.3390/jcm12082959/s1, Table S1. Clinical characteristics of healthy subjects and COPD patients with mMRC grade ≥ 2.
