# Supplementary material for: Computed Tomography Lung Density Analysis: An Imaging Biomarker Predicting Physical Inactivity in Chronic Obstructive Pulmonary Disease: A Pilot Study
Source: J Clin Med. 2023 Apr 19;12(8):2959. doi: 10.3390/jcm12082959 (PMC10146330; doi:10.3390/jcm12082959)
Supplement: Supplementary file 1 [file jcm-12-02959-s001.zip › jcm-2334066-supplementary.pdf]

Table S1. Clinical characteristics of healthy subjects and COPD patients with mMRC grade≥2.

	**HS (n = 12)**	**COPD with mMRC grade≥2 (n = 8)**	**P-value**
Sex (M/F)	6/6	8/0	0.04
Age (year)	62 (56–70)	66 (64–75)	0.18
BMI (kg/m^2)	21.4 (19.8–23.8)	19.9 (19.4–22.5)	0.57
Smoking index (pack-year)	10 (0.0–31)	43 (37–71)	0.008
CAT	4 (3.5–6.3)	13.5 (9.5–22.0)	0.002
mMRC Dyspnea Scale (0/1/2/3/4)	6/6/0/0/0	0/0/5/3/0	<0.0001
FEV_1 (L)	2.65 (2.39–3.16)	1.52 (1.16–2.08)	0.001
FEV_1/FVC (%)	78.2 (75.8–86.9)	54.3 (47.7–64.6)	0.001
FEV_1 % pred (%)	110 (103–115)	52.3 (47.1–70.5)	<0.0001
GOLD stage (1/2/3/4)	-	1/5/2/0	-
FVC % pred (%)	110 (105–120)	88.5 (74.4–105)	0.05
RV % pred (%)	114 (103–117)	132 (99.2–155)	0.44
RV/TLC % pred (%)	100 (90.7–113)	104 (90.3–118)	0.83
IC/TLC (%)	44.4 (42.5–49.6)	33.7 (31.4–38.6)	0.02
%DLco (%)	105 (96.8–125)	77.9 (65.8–90.6)	0.03
%DLco/VA (%)	94.6 (89.9–105)	63.5 (52.4–86.0)	0.07

Data are presented as medians (interquartile ranges). Definition of abbreviations: HS, healthy subjects; COPD, chronic obstructive pulmonary disease; BMI, body mass index; CAT, COPD Assessment Test; mMRC, modified Medical Research Council; GOLD, Global Initiative for Chronic Obstructive Lung Disease; FEV_1, forced expiratory volume in 1 s; FVC, forced vital capacity; RV, residual volume; TLC, total lung capacity; IC, inspiratory capacity; DLCO, carbon monoxide diffusing capacity; VA, alveolar volume.
